# Population Characteristics May Reduce the Levels of Individual Call Identity

**DOI:** 10.1371/journal.pone.0077557

**Published:** 2013-10-29

**Authors:** María del Mar Delgado, Eleonora Caferri, Maria Méndez, José A. Godoy, Letizia Campioni, Vincenzo Penteriani

**Affiliations:** 1 Metapopulation Research Group, Department of Biosciences, University of Helsinki, Helsinki, Finland; 2 Department of Conservation Biology, Estación Biológica de Doñana, CSIC, Seville, Spain; 3 Integrative Ecology Group, Estación Biológica de Doñana, CSIC, Seville, Spain; 4 Finnish Museum of Natural History, Zoological Museum, University of Helsinki, Helsinki, Finland; Universidad Carlos III de Madrid, Spain

## Abstract

Individual variability influences the demographic and evolutionary dynamics of spatially structured populations, and conversely ecological and evolutionary dynamics provide the context under which variations at the individual level occur. Therefore, it is essential to identify and characterize the importance of the different factors that may promote or hinder individual variability. Animal signaling is a prime example of a type of behavior that is largely dependent on both the features of individuals and the characteristics of the population to which they belong. After 10 years studying the dynamics of a population of a long-lived species, the eagle owl (*Bubo bubo*), we investigated the emergence and maintenance of traits that reveal individual identity by focusing on vocal features. We found that individuals inhabiting a high density population characterized by a relative lack of heterogeneity (in terms of prey availability and breeding success) among breeding sites might be selected for reducing the levels of identity. Two non-mutually exclusive hypotheses may explain the structural call patterns we detected: (1) similarity in calls may be principally a consequence of the particular characteristics of the population; and (2) high density may encourage individuals to mimic each other’s vocalizations in a cascade effect, leading to a widespread and unique communication network.

## Introduction

Over the last four decades, the field of ecology has shifted from a phenomenological-based discipline, in which the linkage between an observed pattern and a process in nature is only inferential, to one that structures explanations of population, community, and ecosystem phenomena in terms of underlying mechanisms [1], [2]. This shift, which arose from the importance of considering the role of individual variability in influencing population dynamics, has stimulated an overwhelming number of studies that share the common goal of explaining the extent and the (multi)causality of individual variability (reviewed in [3]).

Individual variability can be generated by differences in environmental conditions or genetic background. Numerous examples of individual variability in life history traits are mentioned in the scientific literature, such as (*i*) the age of maturation [4]–[6]; (*ii*) clutch size [7]; (*iii*) reproductive success [8]–[10]; (*iv*) resting metabolic rates [11]; and (*v*) dispersal strategies [12]–[14]. Animal signaling is a prime example of a type of behavior that is largely dependent on both the features of individuals (e.g. social status, physical condition) and the characteristics of the population (e.g. density, level of fragmentation) to which they belong [15].

In birds, vocalization is one of the main channels for transmitting reliable information about species, sex, or intentions. Vocalizations are usually assumed to encode fitness related information, i.e. through their songs individuals (of both songbirds; e.g. [16], [17], and other species performing calls; e.g. [18], [19]) are able to ‘announce’ their own quality and/or the quality of the territory they occupy. Additionally, numerous observational and experimental studies concerning several bird species have found evidence that vocalization can also reveal individual identity [20]–[22], and different acoustic techniques of individual recognition have been successfully applied to population monitoring. These studies have highlighted the effectiveness of the bioacoustic approach as a non-invasive method for monitoring avian populations [18], [23]–[26]. There is a consensus that the cost of producing song which transmits fitness-related information is only balanced if the environment is heterogeneous and, consequently, when it is really important to discriminate either the quality of the territory or the quality of the owner during vocal signaling [16], [17]. Yet, studies analyzing individual vocal identity have not taken into account the crucial role that the environmental context may play in the evolution and maintenance of traits that reveal individual identity [27].

Here, we study individual vocal identity in eagle owls (*Bubo bubo*) with the aim of addressing an important question: is it possible that a given ecological scenario may reduce the levels of individual call identity? Two previous studies of the eagle owl, a long-lived species characterized by both strong territoriality and pair bonding [28], [29], found that individuals were distinguishable by their calls [21], [30]. Eagle owl vocal behavior is associated with intra- and intersexual territorial disputes, as well as with courtship behavior [28]. We will first characterize our eagle owl study population to demonstrate that it differs considerably from the ones previously investigated [21], [30] with respect to two specific features: (1) the species attains a very high density in our study area (∼40 pairs/100 km^2^; nearest neighbor distance: 250 m; [31]), favoring complex spatio-temporal individual interactions, and (2) individuals live in an environment characterized by high abundance and availability of resources [32], leading to a relative lack of heterogeneity among breeding sites in terms of their quality and productivity. As these two characteristics are not typical attributes of eagle owls [33], [34], the population described in this paper represents an interesting system for the study of particularly unknown aspects of bird vocal communication.

## Methods

### Ethical Standards

Owls were trapped and marked under the Junta de Andalucía–Consejería de Medio Ambiente permit nos. SCFFSAFR/ GGG RS-260 / 02 and SCFFS-AFR/CMM RS-1904 / 02. When the study was performed it was not yet mandatory in Spain to get permission from an ethics committee (legislation: Real Decreto 223/1988). The capture and manipulation of breeding owls posed little risk to the birds given that we immediately removed them from the net, and they remain motionless when manipulated. After eight years of continuous radio-tracking, we have never detected a possible adverse effect that could be directly attributed to the backpacks placed on the birds.

Below we describe an extensive array of methodological approaches used to characterize the eagle owl population (see Table 1 for a short list of abbreviations used). We consider such information to be important for understanding the particular scenario that may be influencing traits revealing the identity of individuals.

**Table 1:** Short list of abbreviations used in the applied methodological approaches.

	Abbreviations	Description
*Acoustic analyses*	CVb	Inter-individual coefficient of variation
	CVi	Individual coefficient of variation
	DFA	Discriminant Function Analyses
	FFT	Fast Fourier Transformation
	Dtot	Total duration of the bouts
	D1	Duration of the portion of increasing frequency
	D2	Duration of the portion of stable frequency
	D3	Duration of the portion of decreasing frequency
	Fmin	Minimum frequency
	Fmax	Maximum frequency
	DOM	Dominant frequency
*Genetic analyses*	H_o_	Observed heterozygosis
	H_E_	Expected heterozygosis
	N_a_	Number of alleles per locus
	Fis	Population inbreeding coefficient
	k	Genetic clusters
	SA	Spatial autocorrelation
	r	Coefficient of autocorrelation

### Data Collection

#### Population parameters

From 2002 to 2012 we studied an eagle owl population located in the Sierra Norte of Seville (37°30′N, 06°03′W, SW Spain; details in [35]). We located 56 nest sites, where a total of 132 breeding attempts were monitored. Laying dates ranged from December 24 to April 8, and the mean (± SD) number of fledglings was 2.18±1.03 per brood (range: 1–4 chicks). Mortality rates were calculated on the basis of 130 radio-tagged individuals (date of first animal tagged: 01/03/03; date of last animal tagged: 22/04/09): mortality of breeders (35.29%; 8 males and 4 females) and dispersers (36.45%; 18 males, 11 females and 4 individuals of sex unknown) were similar. Given the scope of this study, we described the population by means of: (*i*) two measures of productivity, i.e. the mean and the coefficient of variation (CV) of young fledged per breeding pair [34]; (*ii*) an estimate of the quality of breeding sites via census methods of the main eagle owl prey species in the study area, the rabbit *Oryctolagus cuniculus* (mean number of latrines per km of transect ± SE = 20.6±12.4 km^−1^; range: 7.7–46.0 km^−1^); (*iii*) an analysis of the diet through the collection of a minimum of 100 pellets (and as much of prey remains as possible) for each nesting site (mean biomass percentage of rabbit in the diet ± SD = 62.0±19.1%, range = 16–94%; for more details see [32]); and (*iv*) landscape characteristics by intersecting a digital layer representing the boundaries of the owls’ home ranges with a map of landcover elements (scale 1∶25,000). Following the studies of Aebischer et al. [36], and with the aim of selecting only those habitat types that were most relevant for eagle owls [14], [32], we (a) first classified the landscape into 10 landcover types: urban areas, water bodies, forests, dense scrublands with trees, sparse scrub with trees, herbaceous vegetation with trees, scrublands, low vegetation, woody crops and herbaceous plants. Additionally, we used edge density (i.e., the total length of the patch edge per unit area within each landscape; [37]) as a proxy for the effect of habitat heterogeneity [38]–[40], which has been shown to be important in determining breeders’ movements and rhythms of activity [41]. Then (b) we performed a compositional analysis to test owl habitat selection (for more details, see [32]). We used ArcView 3.2 (Geographic Information System, GIS) and its extension Patch Analyst [37] for the analyses of landscape characteristics.

Finally, we analyzed the genetic structure of the population by using a set of loci developed for eagle owls, the spotted owl (*Strix occidentalis lucida*) and the lanyu scops owl (*Otus elegans botelensis*). We extracted DNA, following a Hotshot protocol [42], from blood samples (2 mL, taken from the brachial vein by V.P., who was initially accompanied and trained by an expert veterinary; date of first animal sampled for DNA: 01/03/03; date last animal sampled for DNA: 22/04/09) of 22 adult individuals in our study population. Blood samples were collected under the Junta de Andalucía–Consejería de Medio Ambiente permit nos. SCFFSAFR/ GGG RS-260 / 02 and SCFFS-AFR/CMM RS-1904 / 02. Based on polymorphism of the loci, we finally selected the following 10 loci: Oe3-7, Oe045, Oe054, Oe128, Oe2-57 (GenBank accession no. AY312418, AY312422, AY312425, AY312427, AY312420, respectively) [43]; Bb42, Bb126, Bb131 (GenBank accession no. AF32093, AF32097, AF32098, respectively) [44]; 15A6 and 13D8 [45]. Fluorescently-labeled PCR products were amplified in a reaction with a final volume of 20 µl, which included 50–80 ng of DNA, 67 mM of Tris-HCl, 16 mM of (NH_4_)_2_ SO4, 2 mM of MgCl_2_, 0.2 mM of dNTPs, 0.1 ng/µl of bovine serum albumin (BSA, Biomol), 0.5 µM of reverse and fluorescently-labeled universal M13 primer, 0.041 µM of forward primer and 0.5 units of Taq polymerase (BIOTAQ, Biomol). Reaction conditions were as follows: an initial denaturation step of 2 min at 94°C, 30 s at 55°C annealing temperature decreasing 1°C/cycle for 15 cycles, and 30 s at 72°C, followed by 27 additional cycles with an annealing temperature of 40°C and a final step of 5 min at 72°C. Products were analyzed on an ABI PRISM® 3100 DNA Genetic Analyser (Applied Biosystems) and alleles were scored with GeneMapper 4.0 (Applied Biosystems, Inc.).

#### Individual parameters

We trapped and radio-tagged 34 breeding individuals (24 males and 10 females) from 24 nests, as well as 96 juveniles (54 males and 42 females) from 21 different nest sites (for more details about the radio-tracking procedure see [14], [32]). Each individual was fitted with a 30 g radio-transmitter using a Teflon ribbon backpack harness (Biotrack Ltd, Wareham, BH20 5AJ, Dorset, UK; www.biotrack.co.uk). The mass of the backpack was less than 3% of the mass of the smallest adult male (1550 g; mean ± SE = 1667±105 g) in our population. This telemetry study allowed us to collect detailed information at the individual level concerning both the dispersal process [14], [31] and the breeders’ home ranging behavior [32]. Radiotracking data were analyzed under the framework of animal movement analyses (see [14], [31]–[32] for more details). We found dispersal distances to be very short in most cases, ranging from 1.5 to 34.3 km (mean ± SD = 6.0±4.2 km). In fact, 35% of the individuals which dispersed established a stable range close to their natal population. In general, breeders showed high site fidelity; their home range behavior being simultaneously affected by different internal and external factors acting at different spatio-temporal scales. However, we also recorded nine cases of breeding dispersal (5 males and 4 females), as well as ten cases of replacement of a breeder (5 males and 5 females).

The individual monitoring of this nocturnal species is extremely demanding, especially considering the intrinsic difficulties and relatively low success rates of breeder trapping. Given that two previous studies [21], [30] showed that eagle owl vocalizations are individually distinctive, we were expecting to be able to recognize, over the course of a year, each territory owner within our population by the characteristics of its call sonograms. This procedure would have also favored the use of a technique less intrusive than breeder trapping, i.e. the individual discrimination by territorial and sexual call recording of breeders. Thus, from 2002 to 2006 we recorded 15 males and 10 females at 15 breeding sites using a Sony digital audiotape recorder (TCD-D100) and a Sennheiser directional microphone (condenser microphone ME 67+ powering module K6). Some individuals were recorded over different years, namely six males from the 15 breeding sites that were also captured and radio-tagged. The characteristics of the territorial call of eagle owls are well described in [21].

We strictly followed a rigorous recording protocol. (1) Recordings were always made at sunset for birds positioned on known call posts in close proximity to their nests [46] during calm days (without wind or rain), and the observer was never too far from the birds (less than 100 m). Recordings were made during the pre-breeding period (i.e. September–December in our study area), when males and females are in general more vocally active [28], [47]. (2) Recordings were performed by the same two observers (V.P. and M.D.). (3) We were helped by an expert (P.L.; see acknowledgement) who has a great knowledge of bird recordings and sound analysis. Therefore, we are confident that we carried out a well-designed recording of the breeders in our population, where recorded information was combined with data from radio-tagged individuals, when possible.

**Figure 1:**
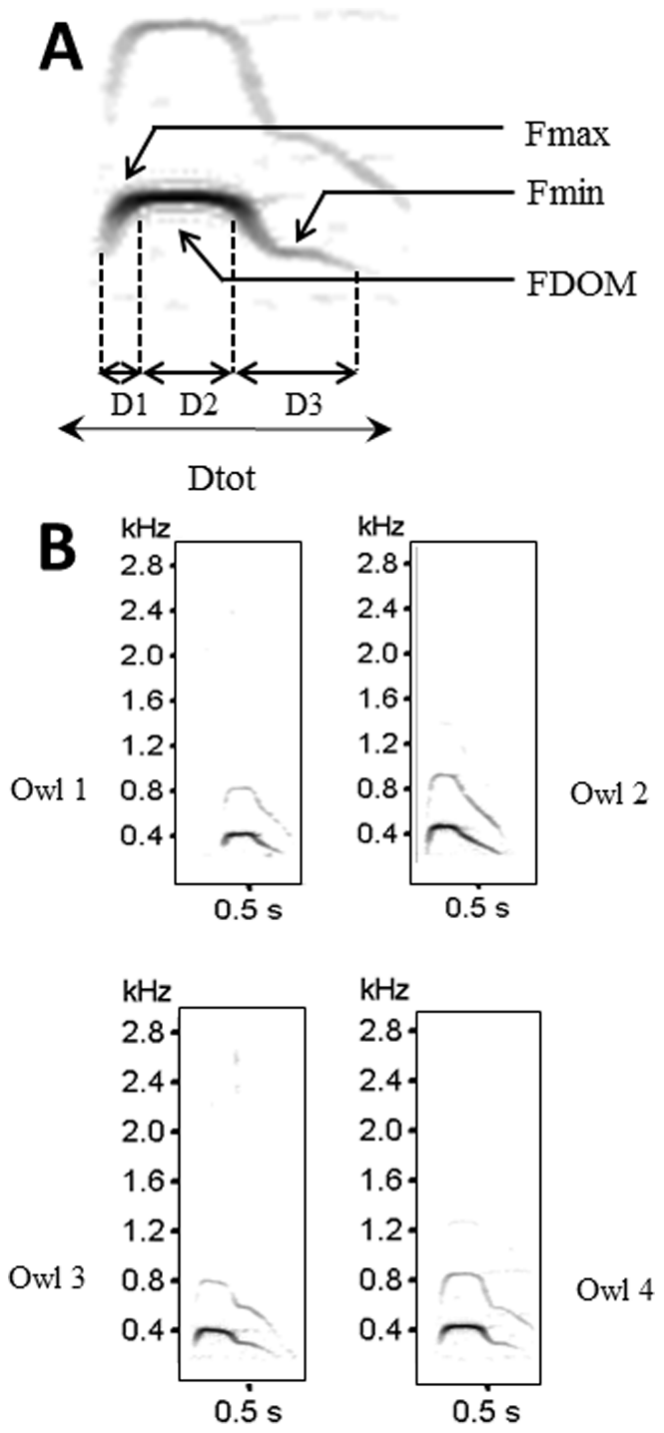
Graphical representation of the spectrograms of the hooting of the eagle owl (A) of male (below) and female (above) calls of the eagle owl. Parameters measured to characterize the call are: (*a*) parameters in the time domain: D1, D2 and D3; (*b*) parameters in the frequency domain: Fmax, Fmin and FDOM (see text for explanations). (B) Four spectrograms of the territorial calls uttered by different eagle owl males in south-western Spain. The high similarity among eagle owl calls is apparent even by visual inspection. Owing to the considerable overlap observed, individuals could not be discriminated on the basis of the information concerning their vocalization.

We extracted the acoustic features of the 478 calls that were recorded on audiotape by performing a spectrographic analysis. For this analyses, we used Avisoft SASLab Pro software (Version 3.91; [48]), performing a Fast Fourier Transform (sampling frequency 11,025 Hz, FFT length 512, time resolution 8.9 ms, bandwidth of frequency resolution 43 Hz, Window Function: Bartlett). For both male and female calls, four temporal variables were measured (Fig. 1A): total duration of the bout (Dtot), duration of the portion of increasing (D1), stable (D2) and decreasing (D3) frequency. Four frequency variables were also measured (Fig. 1A): minimum and maximum frequency (Fmin and Fmax), dominant frequency (DOM; i.e. the frequency with the highest energy) and the range of frequencies (range = Fmax-Fmin) in a bout.

### Data Analysis

#### Population structure analyses

Following Penteriani et al. [34], we analyzed the spatial structure of the population (i.e. population heterogeneity) using several procedures. First, to test the effect of breeding site quality on overall population fecundity, we eliminated the year effect on productivity. Owing to the existing annual variations, we controlled for the year effect by subtracting annual mean from the row data. For the number of fledglings, negative values indicate a poorer breeding performance than average, whereas positive values indicate a better one. Relative productivity was analyzed by a general mixed model, with the breeding site as a random factor to correct for pseudoreplication. Second, we tested a variable designated % of contributing pairs, which allowed us to detect intrinsic variability of the population through the evaluation of the distribution of fecundity among nesting territories. Our assumption was that a heterogeneous population structure, characterized by differences in quality among breeding sites, should lead to low variance in production of young during good years and high variance during poor years (i.e. a few pairs will produce the majority of the fledglings). To accomplish this, we considered the percentage of breeding pairs producing at least 50% of the annual fledged young. We calculated this parameter by summing the number of fledged young (starting from the pairs with highest productivity) necessary to attain 50% of the annual young production. Finally, to detect whether landscape structure, diet (rabbit biomass) and resource abundance explain differences in mean reproductive output and its annual variance within the population, we ran two multiple regression models using (a) mean number of fledglings and (b) CV as dependent variables. We used the open-source software R, version 2.10.1 [49] to build the linear models. We always explored the residuals for: (i) normality, (ii) homogeneity of variance, and (iii) spatial independence. For the latter, we used the package Gstat [50] to verify the independence of the data by plotting the residuals versus their spatial coordinates; the resulting bubble plot did not show any spatial pattern. All tests are two-tailed, statistical significance was set at α <0.05, and ± deviations for means are either SD or SE, depending on whether the factor of interest was variability or precision, respectively.

#### Population genetic analyses

Genetic diversity, i.e. observed (*H_O_*) and expected heterozygosity (*H_E_*), mean number of alleles per locus (*N_a_*), and the population inbreeding coefficient (*F_IS_*), was estimated for each locus for the population using FSTAT. Significance of *F_IS_* was determined by bootstrapping over loci to obtain a 95% confidence interval based on 10,000 replications. The same program was used to perform tests for Hardy-Weinberg equilibrium (HWE) using 10,000 permutations of alleles among individuals. Sequential Bonferroni corrections were applied to correct for multiple simultaneous comparisons. To analyze the genetic structure of the population we used two approaches. First, we used Structure v.2.2 software [51] to assess the number of different genetic clusters (k) in the population. Simulations were run with a burn-in period of 20,000 followed by an additional 2×10^6^ MCMC steps. The number of populations was varied from1 to 5, and for each k 20 replicates were run under an admixture model with correlated gene frequencies. We assessed the support for *k* populations based on visual inspection of the plot of the algorithm of the posterior density (lnP (D)) as a function of *k*, and Δ*k*, following [52]. Convergence was assessed by checking that the posterior density and the log-likelihood levels reached a plateau before the end of the MCMC runs. Second, by using GenAlEx and following the method proposed by [53], we investigated the genetic spatial autocorrelation at the individual level within this population (SA). This analysis allowed us to determine whether related individuals were clustered in space, which might suggest that dispersal is limited by distance, even within the same population. We used a pairwise geographical distance between individuals calculated as the linear distance separating them based on their breeding location, and a pairwise genotypic distance. We estimated the average genetic similarity between pairs of individuals in specific distance classes (thresholds at 500, 1000, 1500 and 2000 m) through the autocorrelation coefficient (r) obtained from 9,999 permutations.

#### Individual acoustic analyses

To identify the presence of sound information concerning individuality [30], [20], [21], we first performed a nonparametric analyses of variance (Kruskal-Wallis ANOVA) to identify the characteristics of calls for which inter-individual variation was higher than intra-individual variation. We assumed that a much greater inter-individual value indicates a factor which is better able to describe individual variation. As a measure of call individuality [30] we also estimated for each variable the ratio between the inter-individual coefficient of variation (CVb) and the individual coefficient of variation (CVi). Once these variables were identified, we performed a discriminant function analysis (DFA) on standardized data [24], [25] to test for the discriminant power of the acoustic features. For this analysis, we used only the calls of the six recorded and radio-tagged males whose identity was known (as in [20]). For classification purposes, we finally applied similarity techniques to define threshold values of similarity within individuals, i.e. calculating the Euclidean distances between the acoustic features of pairs of birds. Following previous studies [20], [25], when a new recorded bird fell outside the intra-individual threshold for all marked birds, it was classified as a new individual. As those birds whose identity was known were all male, we performed classification analyses for this sex only. Discriminant function analysis (DFA) and Euclidean distance estimations were performed with SPSS (version 20). Finally, we used regression analysis to explore whether acoustic similarity was higher for closer neighbors. In fact, because neighboring birds can form local communication networks [54] and match their songs to those of their neighbors [16], a change of vocalization structures and acoustic matching over distance was expected to occur.

## Results

### A Population Characterized by Its Stability and High Fecundity

**Figure 2:**
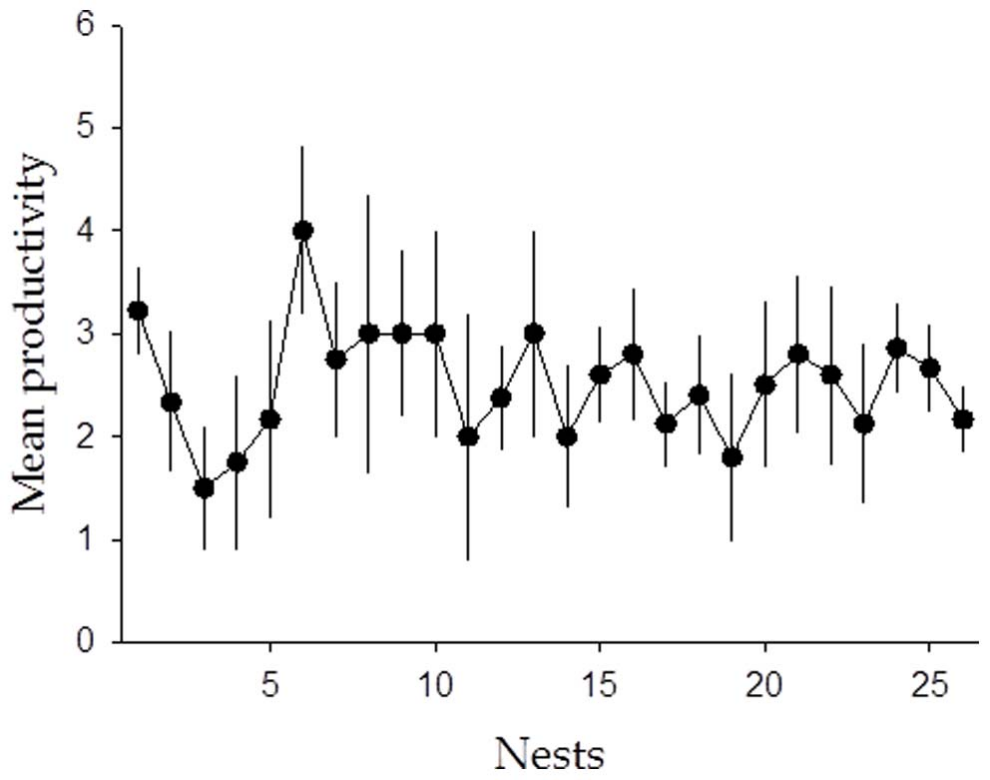
Pattern of mean productivity (±95% CI) of eagle owl territories during a 10-year period. Differences in the fecundity distribution between territories were very small, indicating a relatively homogeneous population, which is characterized by territories of similar quality.

The occupancy rate was very high over the years, i.e. the only context in which we did not find any evidence of reproduction was when a pair disappeared (e.g. one or both members of a breeding pair died), and breeding pairs always reproduced successfully (Fig. 2). After controlling for year effect, no significant differences among territories were detected for productivity (estimate ± SD = −0.0013±0.0097, df = 29, *t* = −0.14, *p* = 0.89; Fig. 2). Moreover, when considering the mean percent of contributing pairs as a threshold to separate good from poor years, more pairs contributed to the production of young during good years (49.82±1.98%) than poor years (40.84±3.59%), but the difference between the number of pairs was only marginally significant (*t* = −4.03, *p* = 0.04). Finally, there was no effect of landscape structure, diet and resource abundance on either mean reproductive output or its CV (for all *p*>0.05). All these results provide evidence for a relatively homogeneous population, which is characterized by territories of similar quality showing rather similar annual variance in productivity.

### A Lack of Significant Differences in Genetic Structure

All markers analyzed were polymorphic in the study population with the observed number of alleles ranging from two (Bb131) to 15 (Oe2-57), with an average of 6.1. Genetic diversity was moderate with an average observed heterozygosity of *H_E_* = 0.675 (range 0.304–0.918). The inbreeding coefficient was high and significantly positive for two of the analyzed markers (Oe2-57: *F_IS = _*0.211, *p* = 0.005; 13D8: *F_IS = _*0.515, *p* = 0.001), suggesting the occurrence of null alleles at these loci. Overall inbreeding was high and significant when these loci were included (*F_IS = _*0.129, *p* = 0.001), but low and not significant when they were removed (*F_IS = _*0.05, *p* = 0.119). Genetic analysis in Structure supported one panmictic population with no significant genetic structure. That is, lnP (D) was highest for k = 1. Moreover, the intra-population analysis at the individual level detected no signal of spatial genetic autocorrelation in any of the distance classes analyzed, nor in the dispersal distance class (1500 m).

### Individual Vocalizations: the Loss of Distinctiveness

All the studied acoustic parameters appeared useful for individual identification, as they exhibited CVb/CVi ratios greater than 1 (ratios ranging from 1.54 to 2.90; Table 2). However, considering that the univariate analysis showed that only six out of the eight parameters initially considered presented highly significant differences between individuals in both male and female groups (Table 2, *p*<0.01), we conservatively decided to only select them for the following multivariate analysis. We entered these six acoustic variables into a DFA that correctly classified 95.8% of vocalization bouts to the marked individual from which they were recorded. The first 5 discriminant functions explained 69.8% of overall data variation and had eigenvalues = 11.2, Wilks’ Lambda = 0.002 and *χ^2^* = 301.71 (*p*<0.001). The maximum value for acoustic (Euclidean) intra-individual distances of known birds was 130.0. However, when using this value as the acoustic threshold of similarity, nearly all of the Euclidean distances between the acoustic features of pairs of unknown birds fell below the intra-individual threshold, in both the same (91.85%) and different (92.46%) years. Therefore, even though such acoustic variables showed some inter-individual variation, they were not able to discriminate between the different eagle owls (see Fig. 1B for an example of the visual comparison between sonograms of different individuals). In addition, we did not detect any acoustic similarity for closer neighbors (*F* = 0.2407, df = 663, *p* = 0.624), as would be expected if communication were limited by distance or if birds matched their songs to local neighbors. The lack of significant differences observed between the calls of eagle owls in our study population did not allow us to discriminate individuals based on the information concerning their vocalizations.

**Table 2:** Characteristics of the temporal and frequency parameters measured from recordings of eagle owl calls (N = 478).

	Dtot	D1	D2	D3	Fmin (Hz)	Fmax (Hz)	DOM (Hz)	Range (Hz)
	♀ ♂	♀ ♂	♀ ♂	♀ ♂	♀ ♂	♀ ♂	♀ ♂	♀ ♂
Mean	0.68 0.56	0.06 0.06	0.24 0.22	0.05 0.06	346.52 222.02	593.12 447.30	534.82 391.87	246.59 225.28
SE	0.005 0.005	0.004 0.0007	0.003 0.002	0.001 0.0006	4.48 1.88	3.79 1.89	3.94 1.80	5.24 2.17
Median	0.68 0.58	0.05 0.05	0.25 0.22	0.05 0.05	370 230	580 440	530 390	240 210
Min	0.50 0.33	0.03 0.03	0.12 0.11	0.02 0.03	230 160	510 370	440 320	140 140
Max	0.82 0.80	0.29 0.11	0.32 0.34	0.09 0.09	460 370	670 580	630 530	400 330
CVb	–	0.21	0.17	0.24	–	0.10	0.09	0.16
CVi	–	0.13	0.08	0.11	–	0.03	0.04	0.07
CVb/CVi	–	1.54	1.99	2.17	–	2.90	2.46	2.07

## Discussion

Our results demonstrate that the levels of individual call identity in our population were low. This finding is contrary to the results presented in a number of previous studies that, instead, clearly showed the existence of a specific individual signature in the vocalizations of many different species (e.g. [25], [26]).

The reduction of an individually distinctive vocal signature may arise because (a) the natural vocal variation within individuals over time is high, leading to levels of ambiguity in the identification of an individual [55], [56]; or (b) the variation between individuals is small [26]. By looking at the values of the coefficient of variation within and between individuals in our population study, we can conclude that the decrease of individual distinctiveness may be attributed to the similarities between individuals in their vocalizations rather than possible variations within individuals over time. In fact, the variation between individuals observed in this study (ranging from 0.09 to 0.24) was negligible compared with that reported by Lengagne [30], who found values between 7.1 and 42. The abovementioned values from these two studies are directly comparable, as they were estimated from similar acoustic parameters.

Following the idea stressed by Tibbetts and Dale [27] about the important role that social and environmental context can play in the evolution and maintenance of traits that reveal individual identity, we hypothesize that the decrease of individual distinctiveness in vocalizations may be attributed to the peculiarities of the study population. In our opinion, two main factors may have determined the similarity in call structure: (1) the population density and (2) the relative lack of heterogeneity (in terms of prey availability and breeding success) among breeding sites. First, the density of the population under study is among the highest ever reported for the species (but see also [57]). Densities were lower for the populations (2.5 pairs/100 km^2^; minimum distance between two recording sites ca. 5 km; [21], [30]) for which it has been possible to distinguish individuals by features of their calls. Second, our long-term study showed that (*i*) fecundity was relatively high and rather identical for the whole population and (*ii*) all pairs successfully bred every year. These two features are not typical attributes of eagle owl populations, which instead are usually characterized by their heterogeneity in quality and fecundity among breeding sites [33], [34]. Actually, prey availability is extremely high in the whole study area [32], which may explain both high density and fecundity. In addition, (*iii*) genetic results showed that there is no genetic structure between individuals in this population: they seem to form one unique panmictic population with no substructure, and with no spatial genetic autocorrelation.

Similarity of eagle owl call types found across the whole range of nest distances might be consistent with a peculiar type of social synchronization of vocalizations between all individuals (not only real neighbors), matching each other and forming a wide communication network [53]. Owing to the unusual high density of breeders and, consequently, the extremely close distances between displaying individuals, a cascade effect between individuals can induce the population to behave as a larger network than the typical one in which only the closest individuals within a population form separated clusters. Actually, the density of breeders in most of the studies of avian networks is much lower, so that each calling individual has just a few neighbors with which to interact and from which to learn [16].

There are at least two biological benefits of the network we identified in this study. A first obvious benefit at the individual level is that possessing similar individual calls can be advantageous during interactions with neighbors. Several researchers have attempted to address the question of why, in numerous territorial species, males interact with neighbors by partially sharing or matching some portion of their song repertoire [16], a phenomenon that has been termed the “dear enemy” effect [58]. Neighboring territorial animals are often intense rivals; however, many studies have found that territorial birds may respond less aggressively toward neighbors than to strangers by overlapping songs to counter-sing with familiar established neighbors. Reduced aggression toward familiar neighbors, especially in a situation in which all individuals inhabit a habitat that is uniformly good and where there is no apparent reason to compete, may decrease the likelihood of escalated contests whose outcome could involve a threat of takeover and a high risk of injury, particularly in a predatory species that has weapons able to inflict damage during conspecific contests.

A second benefit of the observed communication network is that the reduced aggression toward neighbors may lead to the appearance of a high social stability, i.e. territory owners may decrease boundary disputes by having similar calls. This stability may prevent the attraction of floaters to the area [59]. Actually, floaters can potentially use the detection of social instability as a strategy to establish territories [59]. Before starting the dispersal period, we observed that owlets spend several months under parental care (i.e. a post-fledging dependence period; [60]), providing ample time to learn much about the population and the local area. Social stability might be one of the causes of the low recruitment rate of dispersing individuals to their natal area which we recorded [14]. It is worth noting that the fact that we found a reduced individuality in eagle owl calls does not imply that individuals are not able to recognize each other. In fact, the approach that allows us to describe vocalizations and to identify individuals by their calls (e.g. sonograms) may not be consistent with the manner in which individual birds perceive and recognize each other: more subtle mechanisms may be involved in neighbor recognition.

Recently, Laiolo and Tella [61] highlighted how strong the effect of distance among conspecifics in birds can be, demonstrating that gaps within the individual spatial distribution may hinder cultural transmission of call/song types over distances, resulting in an increased differentiation between those individuals which lack many interactions. Following this line of reasoning, we consider it important to conclude by suggesting that there are two non-mutually exclusive explanations for the structural call patterns we detected: (1) similarity in calls may be principally a consequence of the homogeneous structure of the population; and (2) high density may encourage all individuals to match each other in a cascade effect, leading to a widespread and unique communication network. These two potential scenarios may open new lines of research with the aim of establishing which level - the individual or the population - is the one hindering the emergence of individual variability. Indeed, data on different populations and experimental protocols should be necessary for understanding under what conditions individual identity emerges or is actually hampered, allowing us to make inferences about long-term adaptation at the individual level, and the consequences for populations.

Finally, it is important to stress that evolutionary theory predicts that the amount of genetic variance together with the nature of environmental variability can promote or prevent the evolution of individual variability [62]–[64]. Consideration of the evolution of phenotypic variability, in particular individual traits related to honest signaling of fitness-related information or temporary condition, has opened up stimulating avenues of investigation to enhance our understanding of how individuals adapt to different environments [65]–[67]. Yet, traits revealing individual identity have received little attention thus far. Even though it has been frequently overlooked in ecological and behavioral studies, a decrease of individual call identity may have relevant ecological and evolutionary consequences at the individual and population levels.
